# LGBTQ+ AFFIRMING CARE AND UNDETECTABLE=UNTRANSMITTABLE: EVIDENCE FOR OLDER GAY AND BISEXUAL MEN IN THE US SOUTH

**DOI:** 10.1093/geroni/igac059.166

**Published:** 2022-12-20

**Authors:** Ellesse-Roselee Akre (she/her), Tara McKay, Jeff Henne, Adam Conway, Isabel Gothelf, Nitya Kari

**Affiliations:** Geisel School of Medicine at Dartmouth, Lebanon, New Hampshire, United States; Vanderbilt University, Nashville, Tennessee, United States; The Henne Group, San francisco, California, United States; Vanderbilt University, Nashville, Tennessee, United States; Vanderbilt University, Nashville, Tennessee, United States; Vanderbilt University, Nashville, Tennessee, United States

## Abstract

One of the most significant innovations in HIV prevention is the use of HIV treatment to prevent HIV transmission. This information has been disseminated as the “Undetectable = Untransmittable” (U=U) message. Despite evidence of effectiveness, U=U awareness, belief, and understanding remain limited in some communities. In this study we examine whether having an LGBTQ affirming healthcare provider increases U=U awareness, belief, and understanding among midlife and older gay and bisexual men in the US South, an understudied and underserved population and region where new HIV infections are increasing. We use data from the Vanderbilt University Social Networks Aging and Policy Study (VUSNAPS) on sexual minority men aged 50 to 76 from four Southern US states collected in 2020-2021. We find that only one in four men report prior awareness of U=U, but awareness is higher among HIV-negative and HIV-positive men who have an LGBTQ-affirming provider. Having an affirming provider significantly increases U=U belief and understanding, improves risk perception accuracy, and increases the likelihood of having ever tested for HIV among HIV-negative men. Improving access to LGBTQ affirming healthcare may improve U=U awareness, belief, and understanding, which could help to curb HIV transmission in the US South.

